# Exploring molecular mechanisms of aminoglycoside resistance in *Escherichia coli* MG1655 using the antibiotic resistance growth plate

**DOI:** 10.1038/s41598-026-41386-6

**Published:** 2026-03-04

**Authors:** L.B.L. Cullen, C.J.L. Eldridge, B.P. Jones, G. Forster-Wilkins, S. P. Lawton, M.D. Fielder

**Affiliations:** 1 School of Life Sciences, Kingston University London, Kingston Upon-Thames, London, KT1 2EE UK; 2 Natural History Museum, London, UK; 3 School of Veterinary Medicine, University of Surrey, Guildford, Surrey UK; 4 Centre for Epidemiology and Planetary Health, SRUC School of Veterinary Medicine, Scotland’s Rural College, West Mains Road, Edinburgh, EH9 3JG UK

**Keywords:** Molecular biology, Microbiology, Antimicrobials, Bacteriology, Microbial genetics

## Abstract

The overuse of antibiotics in human and animal health has driven the rapid emergence of multi-drug-resistant bacterial strains. To investigate the molecular mechanisms underlying resistance, we developed the Antibiotic Resistance Growth Plate (ARGP), an experimental evolution tool that imposes a spatiotemporal antibiotic gradient. Using the ARGP, *Escherichia coli* MG1655 populations developed resistance to gentamicin up to 10x MIC within two days. Whole-genome sequencing of wildtype and resistant strains revealed mutations in 16 S rRNA genes and the *fusA* gene. Bioinformatic, phylogenetic, and molecular docking analyses were used to demonstrate the mechanical significance of the *fusA* gene in the molecular pathways of gentamicin resistance in *E. coli* MG1655, where mutations in *fusA* alter EF-G function, disrupting gentamicin’s inhibition of ribosomal translocation. These results highlight the rapidity and specificity of adaptive responses to aminoglycosides and establish the ARGP as a versatile tool for studying the molecular pathways driving antibiotic resistance.

**Supplementary Information:**

The online version contains supplementary material available at 10.1038/s41598-026-41386-6.

## Introduction

Extensive misuse of antibiotics within human medicine and animal husbandry has led to a global rise in antimicrobial resistance and the spread of antibiotic resistance in the environment^1^. Antimicrobial chemotherapy can drive resistance within bacterial populations, resulting in the rapid emergence of multi-drug-resistant strains which diminishes the number of effective antimicrobial agents^2^. Predictions suggest the number of annual deaths caused by antimicrobial resistance could increase to 10 million per year by 2050^3^. This coupled with an antimicrobial discovery void, there is an urgent need for novel classes of antibiotics in drug development pipelines^4–6^. Bacteria can acquire mutations through a range of mechanisms as they replicate^7^. Commonly, these mechanisms occur through spontaneous mutations^8,9^ which can significantly alter an organisms’ susceptibility to antibiotics^10–12^. Understanding these mutation-driven mechanisms of resistance is not only central to clinical microbiology but also to the broader One Health framework, which recognises that antibiotic resistance evolves and circulates across humans, animals, and environmental reservoirs. Improved understanding of resistance development at the molecular level provides a foundation for mitigating its spread across these interconnected systems.

Recently, the development of antimicrobial resistance has been associated with accumulations of resistance mutations, a process that can be accelerated under antibiotic selection pressure, which can favor the emergence of hypermutator strains^13–15^. Experimental evolution studies facilitate the study of such strains through enabling the rapid development of resistance mutations over shorter timescales in a controlled environment in vitro.

The study of drug resistance traditionally relied upon the application of antimicrobial chemotherapy as a strong selective pressure to determine bacterial survival under fixed antibiotic concentrations^16^. The study of antimicrobial resistance by agar selection or disc diffusion permits the selection of single nucleotide polymorphisms (SNPs) with large phenotypic effects^2,17^. The implementation of laboratory forced evolution within this field has provided valuable insights into the genetic mutations underpinning antimicrobial resistance^17,18^. Such experiments utilised passive Mutant Selection Windows (MSW), which excludes multiple mutations appearing under sustained drug selection^19,20^ Likewise, adaptive laboratory evolution (ALE) using serial passage systems frequently induce bottlenecks that affect evolutionary dynamics of bacterial populations^21–24^.

The morbidostat^20^ is an innovative selection device which regulates cell growth by automatically adjusting antibiotic concentrations, in response to antibiotic inhibition. In a similar manner, the microbial evolution and growth arena (MEGA)-plate was used experimentally This system allowed the study of resistance in distinct bacterial populations, employing a highly structured spatiotemporal antibiotic gradient^24^. Although laboratory evolution studies vary in experimental approach, findings have suggested that the pathways to which mutations arise and are acquired are highly structured and reproducible^2,20,23–25^. Aminoglycoside antibiotics display broad-spectrum bactericidal activity through binding to the bacterial ribosome and inhibiting ribosomal protein synthesis^26,27^. Mutations associated with aminoglycoside resistance have been identified within components of bacterial ribosomal complex and translation pathways^27,28^. The current study uses isolates of *Escherichia coli* within a spatiotemporal gradient under gentamicin selection pressure where three isogenic populations were tested in parallel within the ARGP. To determine mechanisms of aminoglycoside resistance in *E. coli*, bacterial cultures isolated from the ARGP were subject to whole genome sequencing and bioinformatic analysis to determine the presence and type of sequence change observed in the mutated isolates.

## Materials and methods

### Strains and culture conditions

All laboratory evolution studies were performed with the drug-sensitive *Escherichia coli* K12 (MG1655), wild-type (WT) strain. Strains were grown in sterile Mueller-Hinton medium, and incubated aerobically at 37 °C, unless otherwise stated. Drug solutions of the aminoglycoside gentamicin were prepared from powdered stocks (Sigma-Aldrich: G4198). Stock solutions of gentamicin (50 mg/L) were prepared in deionized water and stored at 2–8 °C. Following media sterilisation, working solutions were prepared through the addition of antibiotic stock solutions to yield the required concentration.

### Development of resistance within the ARGP

**Fig. 1:**
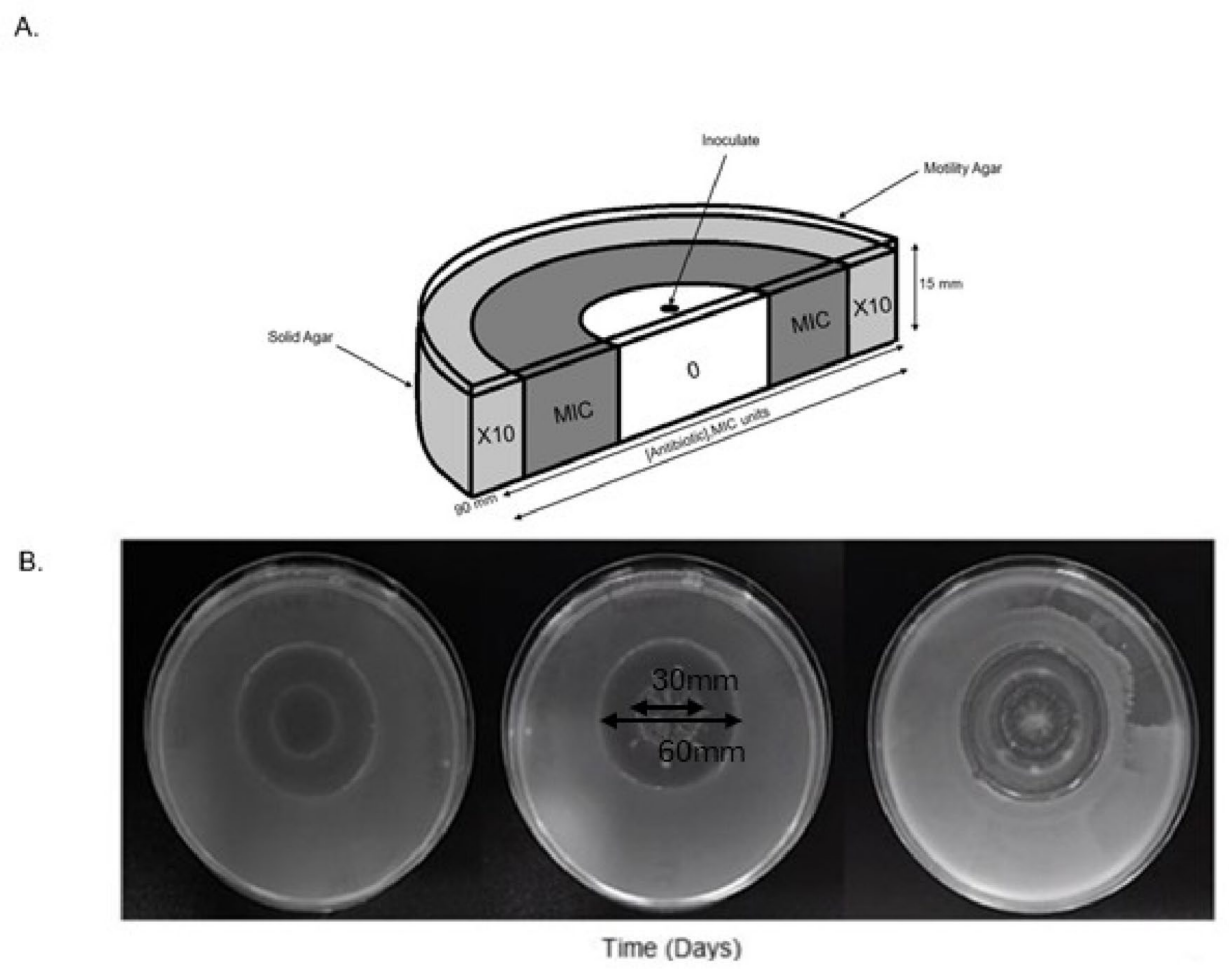
**(A)** The Antimicrobial Resistance Growth Plate (ARGP) set-up. Three-step antimicrobial concentration gradient employed in the ARGP. Antibiotic concentration increasing exponentially by a factor of 10 of the MIC of the selected antimicrobial agent centre circle to the outermost rings of agar. The separate rings were covered with a thin layer of agar to facilitate motility. The plates were inoculated in the central region of the plate that was drug free. Images were taken daily images with the G-Box imaging system. (**B)** Shows the ARGP with a 3% motility layer: with *E. coli* MG1655. G–Box images taken displaying the growth of resistant *E. coli* MG1665 populations exposed to gentamicin selection in the ARGP for a time period of three days. The contrast in these images has been adjusted slightly so as to improve visual acuity but otherwise remain unedited.

The Antibiotic Resistance Growth Plate (ARGP) encompasses a polystyrene dish, 90 mm × 15 mm with spatially structured concentric zones with increasing concentrations of antimicrobial agents. The spatially distinct regions are produced using metallic devices to form circular zones overlaid by a thin motility layer of semi-solid bacteriological agar (3% wt/vol) to enhance bacterial motility (Fig. 1a). Prior to laboratory evolution experiments within the ARGP, the antimicrobial susceptibility profile of *E. coli* MG1655 was established, using the micro-broth dilution method. The wildtype (WT) was selected and defined as the ancestral strain susceptible to the antimicrobial agent gentamicin. The minimal inhibitory concentration (MIC) of the WT, was confirmed as 4 mg/L as defined by the European Committee on Antibiotic Susceptibility Testing (EUCAST). The ARGP was developed with a three-step.

antibiotic concentration gradient with a factor of 10 increase in magnitude per zone of the defined MIC. The intermediate concentrations enable a more gradual growth of resistant strains - more similar to that of a clinical setting. A suspension of the WT (1.5 × 10^8^ CFU/mL), obtained from a freshly prepared overnight culture was then used to inoculate the central antibiotic free region on the ARGP. A total of three parallel isogenic WT cultures had evolved independently within the ARGP per experiment. Bacterial growth was monitored for a period of seven days, contingent on daily confirmatory control experiments for antimicrobial degradation and contamination. Daily images of the ALE experiments were obtained using the G-Box imaging system. Following the appearance of potentially resistant mutational lineages, organisms were sampled from the leading edge of the culture growth. These organisms were dilution streaked onto Muller Hinton agar containing a concentration of antibiotic matching that from where they were sampled. Following 24-hour incubation, aerobically, the resultant isolated colonies were grown for two passages on Muller Hinton agar containing no antibiotics. Isolated colonies were then sampled and inoculated onto Muller Hinton media containing a concentration of antibiotic commensurate with that, that the organism was originally derived. Growth of the passaged isolates on antibiotic containing media indicated that resistance was confirmed having grown on media containing either MIC or 10xMIC levels of antibiotic. Such isolates were cryopreserved prior to genomic analysis.

Any diffusion or degradation of gentamicin was monitored using both NMR and LCMS analysis and found there to be no significant drop in concentration over the experimental period (data not shown).

### Whole genome sequencing and SNP analyses

Whole genome sequencing of the resistant and ancestral strain was provided by Microbes NG. Strains for sequencing were placed into pre-barcoded bead tubes and sent for sequencing facility at Birmingham University. The genomic DNA was extracted, then purified and transferred to an automated liquid handling system (Hamilton Microlab STAR) for quantification, undertaken with the Quantit dsDNA HS assay. Chromosomal DNA libraries were prepared for Illumina sequencing using Nextera XT Library Prep Kit. The pooled library was then quantified before sequencing with a Kapa Biosystems Library Quantification Kit for Illumina on a light cycle 96 qPCR machine (Roche). Whole genome sequencing was carried out using the Illumina HiSeq2000 sequencer with a 250 bp paired ends protocol. The Microbes NG sequencing facility assembled the resistant and ancestral genomes into contigs and provided annotation of their proteomes in GenBank format. GC content for both ancestral and resistant genomes was 50.8%, within the known GC range for *E. coli*.

Strain identification was further confirmed by extracting 16 S rRNA sequences from each genome using Exonerate v2.4.0^29^ and subjecting the sequences to Blastn (https://blast.ncbi.nlm.nih.gov/Blast.cgi). Contigs for the WT and resistant genomes were reordered relative to the reference genome *E. coli* K12 MG1655 (Accession NC_000913, available at https://www.ncbi.nlm.nih.gov/nuccore/NC_000913.3/*)* using Mauve v.2.4.0^30^. An alignment of the reordered WT and resistant strains was subsequently generated in Mauve^30^ and SNPs were identified using the SNP exporter tool. Some SNPs in the bacterial genomes were identified as artefacts of contamination or misalignment via Blastn, as a result we only further explored SNPs from annotated regions which were aligned to the reference genome *E. coli* MG1655. To identify which SNPs were located in annotated tRNA, rRNA or CDS regions we used the snp_ranger.py script available at https://github.com/camilla-eldridge/SNP-Ranger/. SNPs were manually checked for misalignments and nucleotide ambiguity codes by extracting the fasta sequence using Exonerate v2.4.0^29^ (see supplementary Tables 1 and 2 for SNP tables). It was found that some of the genomic regions containing 16 S rRNA genes had not been annotated. As we are specifically interested in the 16 S rRNA genes we used sanger sequencing to re-sequence the 16sRNA regions to identify SNPs. The sequence data supporting the findings of the current study have been placed in the European Nucleotide Archive (primary accession code PRJEB85246).

### Sanger sequencing protocol

For resequencing, we chose to focus on mutations which caused a change either in the amino acid sequence or the RNA sequence of 16rRNA genes. Primer sets were generated using Primer-Blast (https://www.ncbi.nlm.nih.gov/tools/primer-blast/*).* The bacterial DNA of the cryopreserved resistant bacterial populations obtained from the ARGP was extracted through thermal lysis and subject to PCR quality control and amplification before sequencing at the Natural History Museum. The following primers were used for *fusA* gene amplification: fusA-1; 5’-GTGTGGTTAACTCTGGTGAT-3’, fusA-2; 5’-CGCTTCATCATACTTCAGGA-3’ with an expected product size of 1049 bp. For rna123; 5’-CTAACACATGCAAGTCGAAC-3’, 5’-GAATCACAAAGTGGTAAGCG-3’ with an expected product size of 1432 bp and for rna154; 5’-AGAGTTTGATCATGGCTCAG-3’, 5’-CGTTGCATCGAATTAAACCA-3’ with an expected product size of 964 bp. Thermocycling conditions used for all primer sets in the initial gradient PCR reaction were as follows; an initial denaturation step (1 cycle) of 94 °C for 3 min followed by 35 cycles of; denaturation (95 °C for 0.5 min), annealing (52–57 °C for 0.5 min) and extension (72 °C for 2 min). With a final extension cycle of 72 °C for 10 min. The retrieved sequences had DNA sequencing quality assessed by base calling using Tracetuner 3.0.6 from a raw chromatogram using the parameters -Q 3730 and -trim_threshold 20, before quality verification by manual inspection where SNPs with a phred quality score > 25 were confirmed as variants.

### Modelling of *FusA* and *PinR* protein structure

The initial molecular tertiary structure models of the wild-type and mutant proteins were constructed using SCIGRESS^31^. Protein Data Bank (PDB) files were imported into Scigress, and the tertiary structures were superimposed and visualised as solid ribbons for simplicity. Conclusive structure analysis of the mutated proteins was completed using the molecular graphics system PyMOL^32^. Ensuring consistency, all protein PDB files used in this study were rendered using PyMOL with cartoon graphical representations (http://www.pymol.org). Using the integrated python feature, protein domains and proteins within large complexes were selected, highlighted, and manipulated using command syntax and atom selections. In addition, PyMOL was used for the graphical representation of in silico mutagenesis using the PyMOL mutagenesis function. The amino acid residues of the mutant proteins were selected using sequence mode and modelled using wizard mutagenesis.

### Structural analysis of 16srRNA mutations

In the *E. coli* there are seven copies of the rRNA operon. The presence of multiple rRNA operons confers an advantage for increasing the ribosome levels, the key apparatus of translation, in response to environmental conditions. The consensus 16 s rRNA structure of *Escherichia coli* was retrieved from the Java based suite XRNA (http://rna.ucsc.edu/rnacenter/xrna/xrna.html). The 16 s rRNA structure representative of all seven rrn operon variants within *E. coli*, was then annotated to indicate the secondary structure positions of the identified mutations per the reference sequence. The folding of RNA molecules plays an important role in the determination of function^33^. Thus, it was necessary to identify whether the observed rRNA mutations within the given rrn operons resulted in alterations in RNA secondary structure. RNAfold^34^ available at: http://rna.tbi.univie.ac.at/cgi-bin/RNAWebSuite/RNAfold.cgi*)*, which predicts the optimal secondary structure based on the given nucleotide sequence using the dynamic programming technique^35^, was used for the initial prediction and graphical representation of rRNA secondary structures, using default parameters.

The ViennaRNA package 2.0 (http://www.tbi.univie.ac.at/RNA) was subsequently used to for the prediction and analysis of RNA secondary structures based on the relative 16 s rRNA sequences, utilising GGI scripts of secondary structure command line programmes including RNAfold, RNAalifold and RNAinverse^36^. The preliminary 16 s RNA structures were obtained through the RNAfold server, using minimum free energy (MFE) predictions. The RNAfold output consists of the MFE structure in dot bracket notation and graphical representations by means of structures and plots. Adding the options of (–P) and (–MEA) to the command facilitated the calculations of partition function (-P) and maximum expected accuracy (MEA). The RNAplot utility was used to allow the graphical representation of the distinct regions comprising the ribonucleotide mutations. This was achieved using the (mark) command which marks the base of interest followed by the coordinates of the particular base in question. This was then labelled using “label” and at then end of the process “omark” was used to stroke the segment defining a width and a colour inline with the RGB code.

### Molecular docking of the 70s-ribosomal complex with Gentamicin

Computational protein-ligand docking, and virtual drug screening was achieved using the AutoDock suite^37^. Routine protein databases did not carry the protein complex for protein-ligand docking, the crystal structures (pdb.) of the 70s-ribosomal complex in arrangement with Elongation Factor G (EF-G) in both the pre- and post- translocational states was supplied by Professor Jinzhong Lin at the State Key Laboratory of Genetic Engineering at Fudan University in Shanghai, China. AutoDock tools 1.5.2 was used to prepare both the ligand and protein complexes preceding the docking analysis performed within Autodock4.2. To enable molecular docking with the entire ribosomal complex Autodock4 was recompiled with an increased value set for the #define AG_MAX_ATOMS parameter in the constants.h and autogrid.h files.

To ensure the compatibility of the receptor for molecular docking, water residues were removed, followed by the addition of polar hydrogen atoms. The gasteiger charges were then computed and assigned to the receptor and any deficit encountered was spread across residues before merging the non-polar hydrogens. To prepare the antimicrobial compound for docking, the root of the ligand was determined, and all amide bonds were made rotatable before saving the output file in PDBQT format for grid mapping. A grid box size of 60 × 60 × 60 Å points with a spacing of 0.465 Å was considered. Docking was then performed within Autodock using the Lamarckian genetic algorithm. All docked conformations were clustered using a tolerance of 2.0 Å and poses were visualised using the open-source version of PyMOL^32^.

### Phylogenetic analysis of FusA from *Enterobacteriaceae*

The *fusA* gene sequences coding for the Elongation factor G protein obtained from DNA sequencing were subjected to a NCBI nucleotide basic local alignment search (Blastn) (https://blast.ncbi.nlm.nih.gov/Blast.cgi) and aligned with *fusA* gene sequences from the *Enterobacteriaceae* family using MUSCLE^38^. The best fitting substitution model was calculated using JModelTest2 v.2.1.10^39^ as the Jukes-Cantor model based on the lowest value for both AIC (Akaike information criterion) and BIC (Bayesian information criterion) where the lower value indicates a better fit to the data while considering model complexity. EMBOSS Seqret (https://www.ebi.ac.uk/Tools/sfc/emboss_seqret/) was used to convert the DNA alignment to PhyML supported Phylip format (interleaved or non-interleaved). ML trees were generated in PHYML version 3.1^40^ with 1000 bootstrap replicates. The phylogeny was viewed and annotated using iTOL (Interactive Tree of Life) version 4.2 (https://itol.embl.de).

## Results

### Gentamicin resistance evolution in *E. coli using* the ARGP

The ARGP was set-up with a three-step gradient of gentamicin proceeding outwards with a factor or 10 fold increase in MIC (0, 4, 40 mg/L GEN). Gentamicin resistance followed the inoculation of the central drug free regions of the ARGP, with three parallel cultures of the ancestral *E. coli* MG1655 strain. The ARGP facilitated the direct visualisation of resistant populations to X10 the MIC within a period of three days. Despite the consistencies between time to resistance emergence there was significant morphological variation within the spatial temporal gradients. Daily imaging revealed the stochastic nature of resistance development, where following bacterial migration within the antibiotic-free zone, successful resistant lineages emerged as buds often at the extremities of the growth boundaries on the antibiotic containing media (Fig. 1b). The distinct budding of resistant bacterial populations enabled phenotypic sampling of mutants as they appeared within the population.

### Analysis of whole genome sequences

Whole genome analysis initially revealed a total of 145 SNPs between the ancestral and resistant genomes (supplementary Table 1). On further inspection a number of the SNPs were found to be either a result of contamination or mis-alignment. One of the SNPs located on NODE_14 (WT) and NODE_62 (resistant) (Supplementary Tables 2 and 3) appeared to be an artefact of misalignment of the mdoG and mdoC genes between genomes. The SNPs identified in NODE_61 (WT) and NODE_60 (resistant), as well as in NODE_55 (WT) and NODE_55 (resistant) were not located in the annotated CDS region. The SNP in NODE_57 (WT) and NODE_58 (resistant) found in the coding region of the SpoIIIE protein was identified as a *Pseudomonas aeruginosa* protein (99% id and 100% query cover (WP_011274353.1)). The SNP in the protein located on NODE_70 (WT) and NODE_63 (resistant) was inconclusive (supplementary Tables 2 and 3) and could be a result of mis-assembly where the top hit with BLASTp was found to be *Streptococcus pneumoniae* with 93% identity. The only entry in Genbank of this protein for *E. coli* hit with 99% identity for a hypothetical protein, on a partial sequence entry, (WP_160451595). SNPs were identified in the IdrD_2 and YjeO protein coding genes however the mutations were synonymous. Two protein coding genes, fusA, coding for EF-G and PinR, a Serine recombinase family protein, had mutations that changed the resulting amino acid sequence. Some SNPs were identified in genomic regions that originated as a result of misalignment. Only SNPs both identified as *E. coli* by Blastn and were located in annotated tRNA, rRNA and/or CDS regions, corresponding to annotations in the *E. coli* MG1655 reference genome, were studied. A list of SNPs identified in annotated regions can be found in supplementary Tables 2 and 3.

### Mutations in 16 s rRNA identified through re-sequencing

Sanger sequencing of 16 s rRNA identified mutations in two rRNA operons within the 16 s rRNA gene; rna123 and rna154. The differential assembly of 16 s rRNA is critical during 30 S ribosomal subunit formation, owing to precise interactions between 16 s rRNA and twenty ribosomal proteins of the small ribosomal subunit^41^. The presence of 16 s rRNA mutations is expected as gentamicin functions through precise interactions with the 30 S ribosomal subunit^42^. A total of five nucleotide substitutions were identified within the rna123 gene (C208T, G226A, T250A, T253A and A273T) and six substitutions within rna154 (G79A, A80C, T89G, C90T, T93C and G226A). Interestingly, the nucleotide substitution at position 226 was identified in both the rna123 and rna154 genes independently. By contrast, 16 s rRNA sequencing revealed substantial discrepancies in the presence of the rRNA mutations in independent laboratory evolved gentamicin resistant isolates.

### Structural and functional analysis of EF-G

**Fig. 2:**
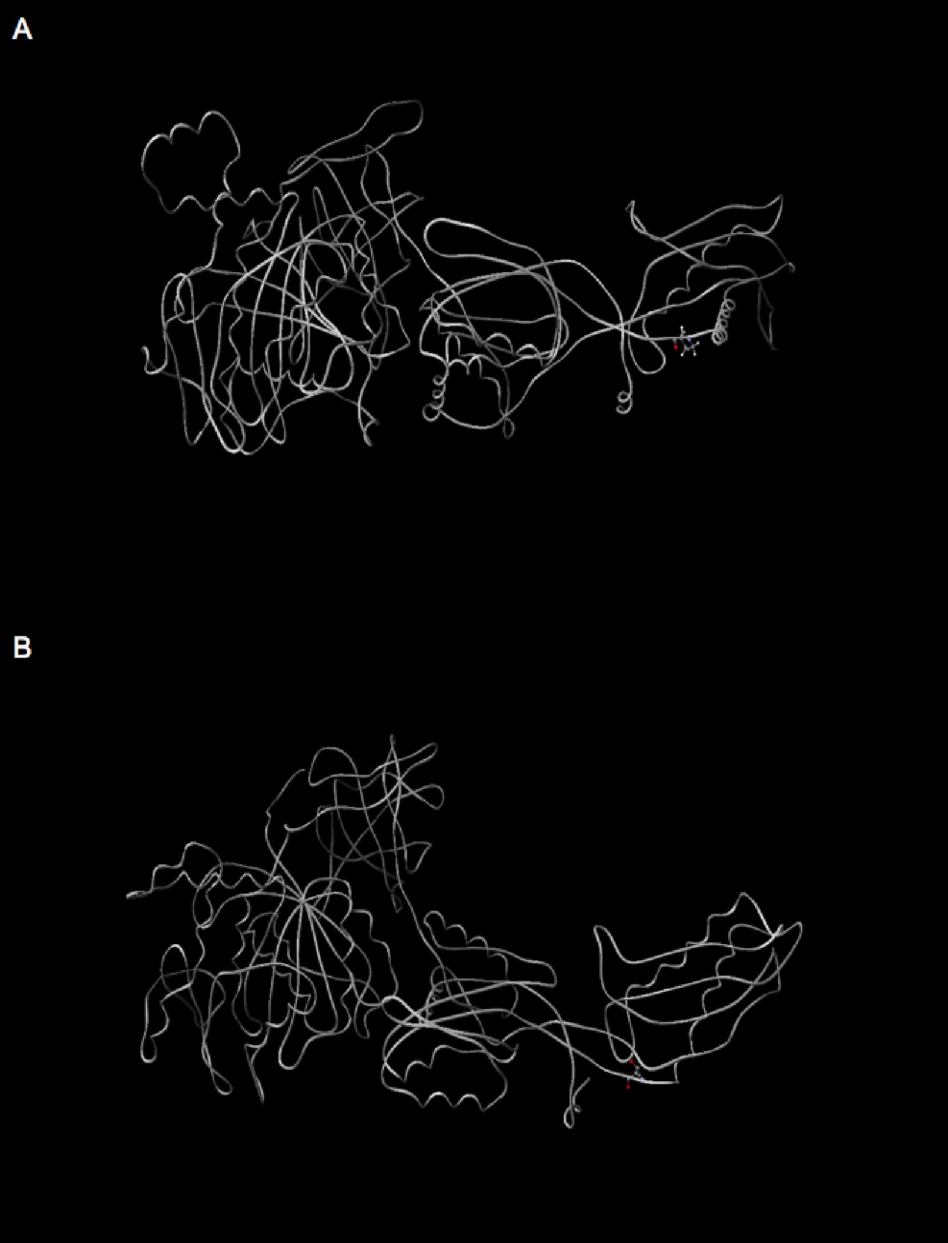
Molecular modelling of EF-G. Scigress was used to show a comparison of EF-G protein models from the WT (**A**) and resistant (**B**) strains of *E. coli* MG1655^31^. Ribbons represent the protein structures whilst the site of mutagenesis is shown by a ball and stick model. Protein compressibility alterations are clear to see between the two protein structures in panel A and panel B at the location of the mutation (shown by the ball and stick model in panel A).

The *fusA* gene encodes EF-G, a protein with GTPase activity which plays an essential role during the translation elongation phase of ribosomal protein synthesis^47^. A single Pro^610^ > Thr^610^ substitution was identified within *fusA*, which was validated based on the recurrence of the SNP in more than one independent gentamicin resistant strain, isolated from the ARGP experiments. The *fusA* encoding EF-G, consists of three superfamilies and a total of five protein domains, allowing the translation factor to operate during two separate phases of ribosomal protein synthesis^43^. The SNP at position 610 was mapped to the EFG_like_IV superfamily between the boundary of domains IV and V. The mutation did not result in any alterations in protein charge, the P610T substitution caused a significant alteration in the protein compressibility and power to be at C-terminus post mutagenesis (*P* < 0.001). This post-mutational modification in protein compressibility was most apparent when modelling the molecular structure of EF-G, where local changes in primary structure appear to have disrupted the overall compactness of tertiary structures of the mutant protein (Fig. 2).

**Fig. 3:**
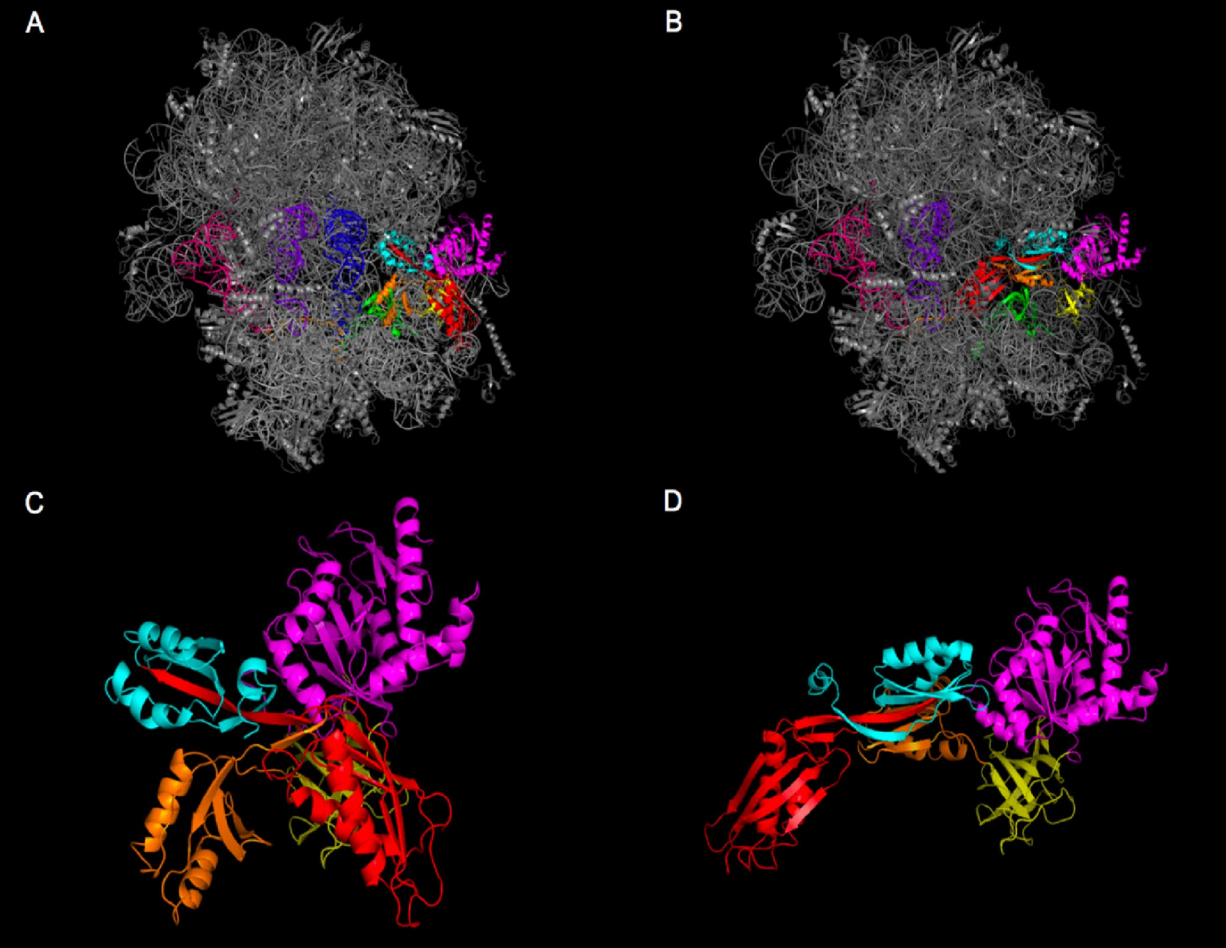
An illustration of the conformational changes observed in EF-G during ribosomal translocation. The pre- (**A**) and post- (**B**) 70s-ribosomal complex as well as the corresponding compact EF-G conformation (**C**) from the pre-complex and the elongated EF-G conformation from the post-complex are displayed.

EF-G undergoes two conformational rearrangements during ribosomal translocation (Fig. 3). EF-G is found in a compacted form during the pre–translocation state (A). With the unoccupied panel A (blue), P (purple) and E (pink) site tRNAs. EF-G experiences a conformational shift to its elongated state occupying the A site tRNA in the post-translocation state (panel B). The peptidyl tRNA moving from the A to the P site and the shifting of the mRNA is coordinated and the change in position is initiated by EF-G, forms the basis of ribosomal protein synthesis^44,45^.

Of the five EF-G domains, domain IV undergoes the most extensive conformational change with its ‘swivel like’ motion. Therefore, it is plausible that mutagenesis at the hinge of domain IV, could interfere with ribosomal translocation. Further Mutational analysis revealed the proline residue located at position 610 is likely essential to the conformational change of domain IV during translocation, as the imino acid has a distinct ability to associate with the protein backbone in two locations and is often associated with polypeptide chains requiring directional change^46^.

###  16 s rRNA functional analysis

**Fig. 4:**
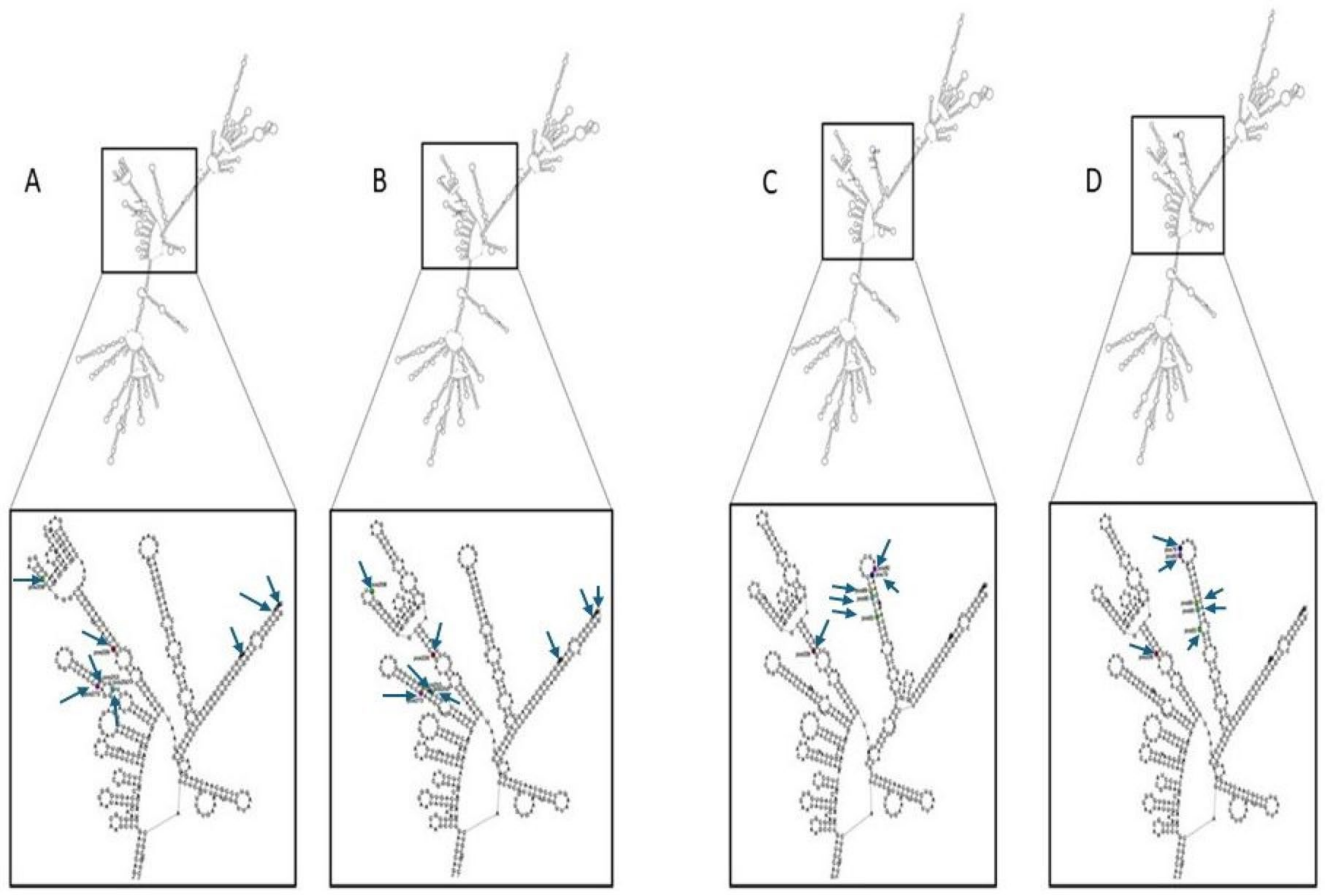
RNA secondary structure analysis of rRNA 123 using Vienna. Displaying the MFE secondary structures of rRNA 123 from WT isolates (in panel **A** and **C**) and the resistant strains (in panel **B** and **D**) of *E. coli* MG1655. The identification of the secondary structure alterations (predicted) between the different MFE structures and the annotation of both the mutational position and ribonucleotide base changes was undertaken with the Vienna package. Arrows are used to indicate the ribonucleotide bases that are mutated in WT and resistant strains.

The initial functional analysis of the 16 s rRNA mutations involved mapping the two rrn operon variants to the consensus secondary structure of *E. coli* rRNA. Despite the independent structural discrepancies between rRNA variants, we pinpointed all nucleotide substitutions to the 5’ body domain of the 16 s rRNA. This is not unexpected as gentamicin binds locally to the ribosomal protein S12, which combines early with the 16 s rRNA in the 5’ body formation during the 30s-ribosomal subunit assembly. We then mapped the mutations to the tertiary structure of the 16 s rRNA, to gain insights into the tertiary interactions of 16 s rRNA within the 30s-ribosomal subunit. Tertiary structure analysis indicates that the rna154 mutations were located posterior to S12 residing within the shoulder of the 30s-ribosomal subunit. The rna123 mutations were predominantly located proximate to domains 1 and V of EF-G. The secondary structures of the independent 16 s rRNA sequence variants were then predicted to detect any local changes in secondary structure induced by the 16 s rRNA mutations. Secondary structure analysis revealed distinctive alterations between the centroid structures of the gentamicin sensitive WT and resistant strains. The secondary structure predictions based on minimum free energy (MFE) appeared highly conserved (Fig. 4), with confined structural changes near the mutated ribonucleotides of the resistant strains of *E. coli* MG1655 (Fig. 4, Panel B and D), when compared to the Sensitive WT strains (Fig. 4, Panel A and C).

### Molecular docking of 70 s ribosomal complex to gentamicin

**Fig. 5:**
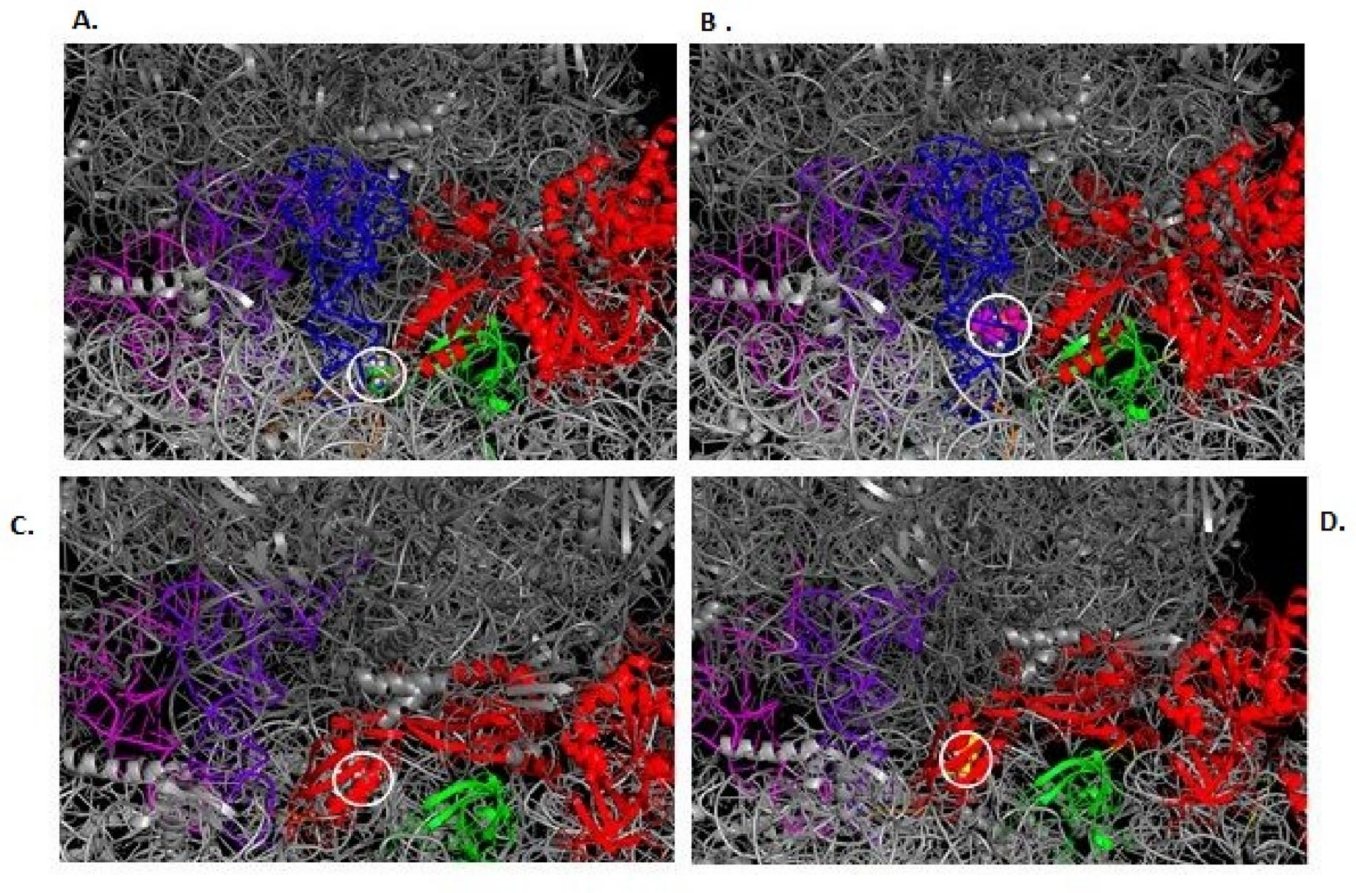
Panel (**A** and **B**) Docking simulation comparison of gentamicin bound to the 70 s pre-ribosomal complex pre- and post-mutagenesis. PyMOL was used to visualise docking analysis and displayed lowest gentamicin binding energy conformation the images are depicted with a space filling model (indicated in a white circle) docking to a 70 s pre-ribosomal complex pre-mutagenesis (A) and in post mutagenesis form panel B. There is a clear altered drug binding post mutagenesis when compared to the gentamicin bound to the 70 s pre-ribosomal complex. This is shown by the gentamicin position in relation to the 30s-ribosomal subunit protein S12 (shown in green), the EF-G (shown in red), the A-site tRNA (shown in blue) and the mRNA sequence (shown in orange). Panel (**C** and **D**) Shows the docking simulation comparisons of the drug gentamicin bound to 70 s post-ribosomal complex in the pre- and post-mutagenesis states. Visualised in PyMOL in the same conditions Docking analysis visualised in PyMOL displaying the lowest gentamicin binding energy conformation depicted using a space filling model (circled in white) docked to the 70 s post-ribosomal complex pre-mutagenesis (panel C) and post-mutagenesis (D). If gentamicin does not dock in the pre-state, it will not successfully bind in a position which leads to the interruption of protein synthesis.

*M*olecular docking simulations were conducted on EF-G bound to the 70s-ribosome in both pre- and post-translocation states (Fig. 5A and B). However, since gentamicin traps EF-G in the pre-translocation state and blocks translocation through the constraints imposed by the A-site tRNA, it was unsurprising that no alterations in ligand binding were observed within the post- ribosomal complex^43^. Comparative docking analysis within the pre-ribosomal complex suggests the P610T mutation of EF-G has implications for gentamicin binding. The results revealed that although docking simulations were comparable in terms of the lowest binding energy conformation of gentamicin (−108.2 K), there were significant changes in the interatomic distances within protein ligand complex pre- and post- mutagenesis (Fig. 5C and D).

### fusA gene evolution in *Enterobacteriaceae*

**Fig. 6:**
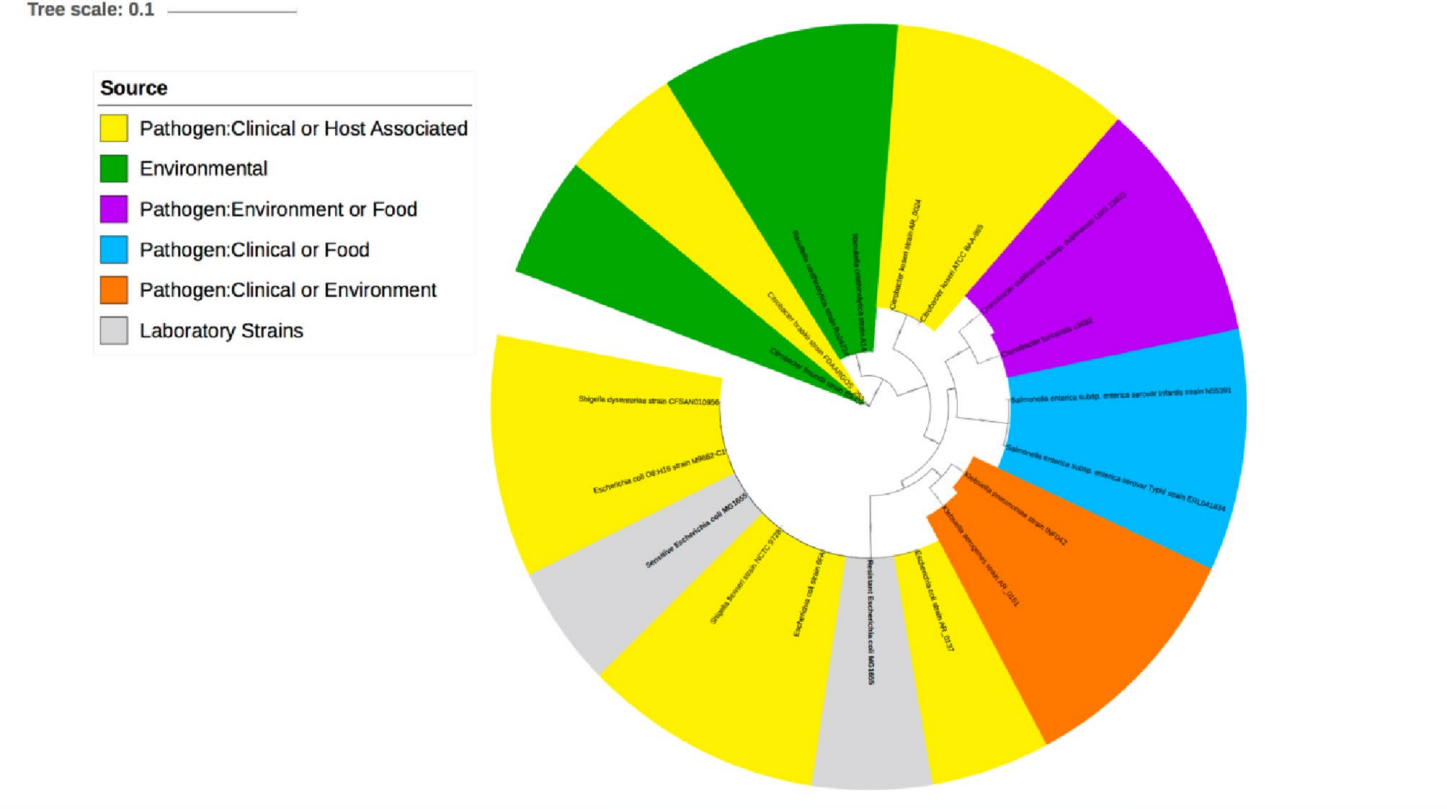
A illustration of distantly related species of the *Enterobacteriaceae* family in relation to the phylogenetic analysis of the *fusA* gene. Showing the *fusA* gene ML tree in distinct species of the *Enterobacteriaceae* family. Analysis carried out using PhyML and the Jukes-Cantor model, the trees were bootstrapped (1000 times). iTOL was used to visulaise the ML tree. Genetic sequences were collected for the *Enterobacteriaceae* species from the NCBI Genbank database employing *E. coli* MG1655 sequences and Blastn in the query. In the ML tree the *Enterobacteriaceae* species are labelled at the branch tips and assigned a colour based on strain locality/source: environmental (green). pathogen clinical/food (blue), pathogen environment/food (purple), pathogen clinical/environment(orange), clinical (yellow) and laboratory (grey). The resistant laboratory strains of *E. coli* MG1655 and WT are highlighted in bold.

A phylogenetic approach was applied to explore the evolutionary history of the *fusA* encoding EF-G gene. NCBI Nucleotide (Genbank) database was utilised as the source for *Enterobacteriaceae* species sequences as it is considered to be a complete, and authoritative, record of publicly available nucleotide sequences. The use of this database was therefore expected to deliver the best selection of *fusA* gene sequences for the phylogenetic analysis when it was carried out. The phylogeny of the *fusA* gene was well resolved within the *Escherichia* genus and the *Enterobacteriaceae* family (Fig. 6) and there was no evidence of evolutionary convergence of the *fusA* resistant mutation within the two taxonomic levels, with the *fusA* mutation localised to a single branch. The tree revealed species clustering of the *fusA* gene, with strong support (Fig. 6). SMART analysis^47^ confirmed that topological disparities between species of the *Enterobacteriaceae* family did not result in any structural alterations within the functional domains of EF-G. These results were reinforced through Praline^48^, which revealed high levels of protein conservation across the *fusA* gene within the *Enterobacteriaceae* family, with distinct regions of species-specific variation. Interestingly analysis also showed maximum conservation at the site of mutagenesis, indicating the biological significance of the proline residue at position 610. Despite this, the P610T mutation has been previously associated with resistance to the aminoglycoside Kanamycin in an experimentally evolved laboratory strain of *E. coli* MG1655^49^, furthermore resequencing of the *fusA* gene in this study confirmed the existence of the SNP in gentamicin resistant colonies. To determine the clinical significance of the resistance conferring mutation, the *fusA* resistant nucleotide sequence was screened at UKHSA against a collection of carbapenemase and ESBL producing *E. coli*. Bioinformatic analysis of 840 carbapenemase-producing and 958 ESBL-producing *E. coli* genomes did not detect the P610T mutation however when subjecting the resistant fusA protein sequence to BLASTp one hit was identified with 100% query cover and 100% identity to a clinical sample from *Homo sapiens* (Accession: HAI7289671.1).

## Discussion

Zhang (a) et al. Zhang (b) et al. and Baym et al. have previously described the use of spatial antibiotic concentration gradients in the study of bacterial evolution^23–25^ and more recently by Ghaddar et al.^50^. There are video representations of this type bacterial growth against an antibiotic gradient produced by Roy Kishony’s group at Harvard Medical School and can be observed using the following link (https://www.youtube.com/watch?v=plVk4NVIUh8). The direct visualisation of bacterial populations within the ARGP enabled the observation of established resistance across antimicrobial gradients. Through subsequent analysis of resistant isolates from the ARGP, using WGS, the underlying genotypic mutational pathways to resistance could be predicted. The ARGP holds potential for exploring how mutational pathways to resistance can vary depending on the antibiotic landscape and strength of these selective pressures. A potential application of the ARGP is within diagnostic experimental evolution, where during complex cases, clinical strains could be subject to a range of antimicrobial agents both independently or in combination, to inform clinical decisions based upon evolutionary risk management^17,51^. In addition, the ARGP tool could be beneficial in determining the resistance potential of novel antimicrobial compounds during the preclinical development phase^52^. More recently, it has been suggested that experimental evolution could be exploited during the modification of pre-existing antimicrobial agents to restrict or reverse antimicrobial resistance evolution^53^. The simplicity of the ARGP provides scope for its application within educational settings, to demonstrate the rapid rates of resistance evolution amongst bacterial species.

The current work describes mechanisms of resistance in *E. coli* MG1655 when exposed to increasing concentrations of the antimicrobial agent gentamicin within a spatiotemporal gradient. The results demonstrate the increase in resistance over time, with evolving bacterial populations achieving resistance to concentrations 10x MIC in as little as two days. Sequencing of the WT and 2 mg/L resistant strain revealed novel mutations in both the 16 s rRNA genes and the *fusA* gene encoding EF-G in the resistant genome, related specifically to antimicrobial mechanisms of action. These observations indicate the mechanism of resistance to gentamicin in *E. coli* is not solely restricted to mutations of the 16 s rRNA target, they can occur more widely through mutations in genes that are highly conserved including *fusA*, encoding proteins that are involved directly in the biological process of ribosomal protein synthesis. Comparable findings of mutations within the elongation factor *fusA* have been identified previously in laboratory evolution studies, suggesting that aminoglycosides elicit similar selective pressures on EF-G within bacterial populations^54–56^. It is well established that aminoglycoside resistance is mediated by mutations in genes encoding the 16 s rRNA^57,58^ Despite the presence of multiple rRNA operons copies within *E. coli*, it is not easy to infer resistance is exclusively mediated by this mechanism^59^.

When looking at the 16 s rRNA mutation database it can be suggested that the mutations identified in this work were not located within domains with high functional importance^60^. However, it is essential that there is an association of the 16 s rRNA with ribosomal proteins for the assembly of the 30s-ribosomal subunit^61^. Mutations within conserved regions of the rRNA binding sites have been shown to impair ribosomal protein recognition and binding affinity^62,63^. Moreover, there are structural requirements of the 16 s rRNA during aminoglycoside binding^64^. Footprinting studies have revealed a protective interaction between streptomycin and concise regions of the 16 s rRNA, which confer phenotypic resistance when mutated by disrupting drug binding^65,66^. Similarly, single rRNA allelic derivatives of *Mycobacterium smegmatis*, hygromycin B resistant mutations were identified in residues in proximity to the binding site of the antimicrobial^67^. Consequently, allosteric interactions occurring between helix 6 and the neighbouring helix 44 of the 16 s rRNA post-mutagenesis could disrupt residues within the gentamicin binding pocket and lead to phenotypic resistance.

Structural alterations within the 16 s rRNA could alter gentamicin binding^68,69^. Demonstrating functional analysis of EF-G is of great importance due to its pivotal involvement in the process of protein synthesis. This occurs via the mediation of mRNA and tRNAs translocation through the ribosome^70^. There is extensive involvement of EF-G in the two autonomous phases of ribosome translocation. There is spontaneous tRNA movement in the initial phase moving between A- and P- ribosomal sites in relation to the 50s-subunit, thus forming the hybrid and classical pre-translocation conformations^71–73^. During this initial phase one important EF-G binding function is to transiently stabilise the intermediate altered (rotated) hybrid state of the pre-translocation ribosome^74,75^. There is EF-G mediated catalysis of mRNA and tRNA movement during the second phase, combined with a reverse rotation of the 30s-ribosome subunit^4,76–78^, which is accelerated through GTP hydrolysis^79^. Early structural studies revealed EF-G contains five structurally defined domains, central to understanding the molecular mechanisms behind ribosomal translocation^80,81^. Domains 3, IV and V have combined functional significance, through the molecular mimicry of the aminoacyl-tRNA at the ribosomal A-site^82^. Interestingly, in its elongated state, domain IV, replicates the anticodon arm of the aa-tRNA. This domains deletion results in translocational activity being diminished^83^. This is compelling evidence to suggest that the mutation identified in the boundary between domains IV and V, could significantly alter the activity of domain IV during ribosomal translocation. When the structural conformations are studied further it is clear that there is substantial rearrangement of EG-F to ensure there is not a steric clash at the ribosomal A-site pre-translocation with domain IV^84–86^. The conformational variability displayed by EF-G in its bound form, is thought to be essential in promoting ribosomal translocation^87^. If, as a result of disulfide crosslinks being added, the intramolecular mobility of EF-G is affected and so it cannot facilitate ribosomal translocation even though GTPase activity is maintained^88^. This is significant, as a result of the replacement of a proline reside (highly flexible) at the location of the molecular hinge in domain IV and the subsequent alterations predicted in reference to tertiary structures and the compressibility of the protein. Proline residues have been recognised for their ability to form molecular hinges, which induce conformational changes within protein structures^89^. Furthermore, mutation induced changes in protein compressibility have been closely associated with modifications in flexibility and overall functional dynamics of proteins^90,91^. Consequently, the replacement of a proline residue at the domain IV hinge, could significantly affect the intrinsic flexibility of EF-G.

Aminoglycoside antibiotics induce so called pleiotropic effects on the ribosome that then disrupt translation by in its pre-translocation complex. This achieved by reducing the tRNA selection accuracy at the decoding site and subsequently and so causing an inhibitory effect on EF-G dependent translocation^88,92^. This observation serves to confirms why alterations are not seen in binding of gentamicin to post-translocation complexes post-mutagenesis. It is known that the activity of an aminoglycoside dependent on the drug binding to the ribosome when it is in its pre-translocation conformation. Helix 44 of the 16 s rRNA major groove is the predominant binding site of aminoglycoside antibiotics, and this is where more precise interactions are formed with decoding bases A1492 and A1493^93^. The changes and alterations that arise following the binding of aminoglycoside to decoding regions, are suggested to have a role in stabilising the classical state of pre-translocation complexes and so increase tRNA affinity to the A-site^94^. In silico molecular docking analysis, predicted gentamicin was unable to bind to the decoding region of the pre-translocation complex following mutagenesis of *fusA* and therefore is unsuccessful in inhibiting ribosomal translocation. This suggests that mutagenesis of *fusA* is the primary mechanism facilitating gentamicin resistance within the in vitro populations of *E. coli* MG1655. Elongation factor EF-G is bacterial protein of some importance in relation to translational GTPases (trGTPases), comprising translation factors that are essential and conserved across pretty much all domains of life^95^. EF-Tu and EF-G The main prokaryotic elongation factors that are involved in two phases of ribosome translation. They have a mutual GTPase activity and they possess some structural similarities that indicate the possibility that they have a common ancestor^96^. Indeed, the fact that EF-Tu is highly conserved means it can be seen as a useful evolutionary marker in bacterial phylogeny, potentially providing phenotypic details missed using conventional 16 s rRNA^97^. Recently, EF-G encoding *fusA* gene was included in Multilocus Sequence Typing (MLST) systems, to discriminate between isolates in the *Cronobacter* genus that show particular diversity^98^. Highly conserved regions in the EF-G protein sequence were revealed using protein conservation analysis and this was consistent over the *Enterobacteriaceae* family. The observation could reflect some functional constraints in the divergent evolution of EF proteins, relative to the proteins essential role during bacterial protein synthesis^99^. Phylogenetic analysis conducted here confirmed a monophyletic origin of the *fusA* gene across all species within the *Enterobacteriaceae* family, further suggesting the functional significance of this highly conserved gene (Fig. 6). Variability within the non-conserved regions is more likely related to speciation events than protein structure or function^100^ Thus, the P610T mutation located within a functionally conserved region of EF-G is expected to drastically alter protein function and phenotype of the organism^101^. Despite antimicrobial concentration gradients incorporated to highlight heterogeneity in natural environments, it isn’t likely that bacterial populations would be exposed to elevated concentrations of an antimicrobial in nature, similar to that utilised in the model^102^. Furthermore, the EF-G mutation was only identified in one clinical strain of thousands, suggesting the mutation may not be common in populations subject to less extreme selective pressures. The model also doesn’t represent all selective pressures facing bacterial populations in natural/clinical settings including fluctuating environmental conditions, bacterial competition and immune interactions^103,104^. It is important that mutational dynamics within bacterial populations are acknowledged, as well as where seemingly beneficial mutations are selected for, other than when other, coexisting populations are present, harbouring a substantial fitness advantage^105^. It is tempting to speculate that the *fusA* mutation identified here might have been eliminated from certain bacterial populations before reaching a detectable level.

The data presented here suggests that gentamicin resistance can arise via mutation in *fusA* and 16 s rRNA genes. The observed P610T mutagenesis of EF-G likely hinders binding of 16 s rRNA targets adjacent to the A-site tRNA, rendering gentamicin incapable of interrupting wobble base formation, leading to termination of ribosomal protein synthesis and ultimately gentamicin resistance. It is essential to build a catalogue of resistance mechanisms for predicting antibiotic resistance strategies that can be exploited by bacterial strains. Future studies will include bacterial pathogens and antimicrobial classes where mutations have the greatest clinical impact^7^. This, coupled with the addition of greater numbers of parallel evolving cultures, under varying selective pressures, would give more accurate representations of the natural environment. The ARGP presented in this work facilitated the identification of previously unknown resistance mechanisms to Gentamicin in *E. coli* MG1655 illustrating that this tool provides a versatile platform for future studies on antibiotic resistance evolution.

## Supplementary Information

Below is the link to the electronic supplementary material.

Supplementary Material 1

## Data Availability

Data is provided within the manuscript or supplementary information files and also on the ENA under project code PRJEB85246.
