# Supplementary material for: Exploring molecular mechanisms of aminoglycoside resistance in Escherichia coli MG1655 using the antibiotic resistance growth plate
Source: Sci Rep. 2026 Mar 4;16:11958. doi: 10.1038/s41598-026-41386-6 (PMC13068909; doi:10.1038/s41598-026-41386-6)
Supplement: Supplementary file 1 — Supplementary Material 1 [file 41598_2026_41386_MOESM1_ESM.docx]

Sequence data supporting the study findings have been deposited in the European Nucleotide Archive with the primary accession code PRJEB85246.

Supplementary Table 1. SNPS exported from MAUVE between WT and resistant strains.

| **SNP** | **NodeID**  **(WT)** | **contig_len** | **coverage** | **pos_in_contig** | **pos_in_genome** | **NodeID**  **(Res)** | **contig_len** | **coverage** | **pos_in_contig** | **pos_in_genome** |
| --- | --- | --- | --- | --- | --- | --- | --- | --- | --- | --- |
| ac | NODE_14 | 114065 | 22.8283 | 1746 | 1062759 | NODE_16 | 112363 | 73.2593 | 1746 | 1060305 |
| tc | NODE_14 | 114065 | 22.8283 | 1786 | 1062799 | NODE_16 | 112363 | 73.2593 | 1786 | 1060345 |
| tc | NODE_14 | 114065 | 22.8283 | 99500 | 1160513 | NODE_16 | 112363 | 73.2593 | 99500 | 1158059 |
| ga | NODE_14 | 114065 | 22.8283 | 112605 | 1173618 | NODE_62 | 1829 | 69.3267 | 222 | 1171144 |
| ct | NODE_14 | 114065 | 22.8283 | 112609 | 1173622 | NODE_62 | 1829 | 69.3267 | 226 | 1171148 |
| ga | NODE_14 | 114065 | 22.8283 | 112611 | 1173624 | NODE_62 | 1829 | 69.3267 | 228 | 1171150 |
| ac | NODE_14 | 114065 | 22.8283 | 112613 | 1173626 | NODE_62 | 1829 | 69.3267 | 230 | 1171152 |
| tg | NODE_14 | 114065 | 22.8283 | 112614 | 1173627 | NODE_62 | 1829 | 69.3267 | 231 | 1171153 |
| tc | NODE_14 | 114065 | 22.8283 | 112615 | 1173628 | NODE_62 | 1829 | 69.3267 | 232 | 1171154 |
| at | NODE_14 | 114065 | 22.8283 | 112619 | 1173632 | NODE_62 | 1829 | 69.3267 | 236 | 1171158 |
| ga | NODE_14 | 114065 | 22.8283 | 112620 | 1173633 | NODE_62 | 1829 | 69.3267 | 237 | 1171159 |
| ct | NODE_14 | 114065 | 22.8283 | 112621 | 1173634 | NODE_62 | 1829 | 69.3267 | 238 | 1171160 |
| ca | NODE_14 | 114065 | 22.8283 | 112623 | 1173636 | NODE_62 | 1829 | 69.3267 | 240 | 1171162 |
| ca | NODE_14 | 114065 | 22.8283 | 112624 | 1173637 | NODE_62 | 1829 | 69.3267 | 241 | 1171163 |
| gt | NODE_14 | 114065 | 22.8283 | 112625 | 1173638 | NODE_62 | 1829 | 69.3267 | 242 | 1171164 |
| tc | NODE_14 | 114065 | 22.8283 | 112628 | 1173641 | NODE_62 | 1829 | 69.3267 | 244 | 1171166 |
| ta | NODE_14 | 114065 | 22.8283 | 112629 | 1173642 | NODE_62 | 1829 | 69.3267 | 245 | 1171167 |
| ag | NODE_14 | 114065 | 22.8283 | 112630 | 1173643 | NODE_62 | 1829 | 69.3267 | 246 | 1171168 |
| ac | NODE_14 | 114065 | 22.8283 | 112634 | 1173647 | NODE_62 | 1829 | 69.3267 | 250 | 1171172 |
| ca | NODE_14 | 114065 | 22.8283 | 112636 | 1173649 | NODE_62 | 1829 | 69.3267 | 252 | 1171174 |
| tg | NODE_14 | 114065 | 22.8283 | 112638 | 1173651 | NODE_62 | 1829 | 69.3267 | 254 | 1171176 |
| tg | NODE_14 | 114065 | 22.8283 | 112639 | 1173652 | NODE_62 | 1829 | 69.3267 | 255 | 1171177 |
| tg | NODE_14 | 114065 | 22.8283 | 112640 | 1173653 | NODE_62 | 1829 | 69.3267 | 256 | 1171178 |
| ag | NODE_14 | 114065 | 22.8283 | 112641 | 1173654 | NODE_62 | 1829 | 69.3267 | 257 | 1171179 |
| ag | NODE_14 | 114065 | 22.8283 | 112642 | 1173655 | NODE_62 | 1829 | 69.3267 | 258 | 1171180 |
| ag | NODE_14 | 114065 | 22.8283 | 112643 | 1173656 | NODE_62 | 1829 | 69.3267 | 259 | 1171181 |
| ct | NODE_14 | 114065 | 22.8283 | 112645 | 1173658 | NODE_62 | 1829 | 69.3267 | 261 | 1171183 |
| tc | NODE_14 | 114065 | 22.8283 | 112649 | 1173662 | NODE_62 | 1829 | 69.3267 | 265 | 1171187 |
| ac | NODE_14 | 114065 | 22.8283 | 112652 | 1173665 | NODE_62 | 1829 | 69.3267 | 268 | 1171190 |
| ac | NODE_14 | 114065 | 22.8283 | 112655 | 1173668 | NODE_62 | 1829 | 69.3267 | 271 | 1171193 |
| ct | NODE_14 | 114065 | 22.8283 | 112657 | 1173670 | NODE_62 | 1829 | 69.3267 | 273 | 1171195 |
| ac | NODE_14 | 114065 | 22.8283 | 112658 | 1173671 | NODE_62 | 1829 | 69.3267 | 274 | 1171196 |
| gt | NODE_14 | 114065 | 22.8283 | 112661 | 1173674 | NODE_62 | 1829 | 69.3267 | 277 | 1171199 |
| gt | NODE_14 | 114065 | 22.8283 | 112662 | 1173675 | NODE_62 | 1829 | 69.3267 | 278 | 1171200 |
| gt | NODE_14 | 114065 | 22.8283 | 112665 | 1173678 | NODE_62 | 1829 | 69.3267 | 280 | 1171202 |
| ga | NODE_14 | 114065 | 22.8283 | 112666 | 1173679 | NODE_62 | 1829 | 69.3267 | 281 | 1171203 |
| ta | NODE_14 | 114065 | 22.8283 | 112669 | 1173682 | NODE_62 | 1829 | 69.3267 | 284 | 1171206 |
| ac | NODE_14 | 114065 | 22.8283 | 112670 | 1173683 | NODE_62 | 1829 | 69.3267 | 285 | 1171207 |
| ga | NODE_14 | 114065 | 22.8283 | 112686 | 1173699 | NODE_62 | 1829 | 69.3267 | 299 | 1171221 |
| cg | NODE_14 | 114065 | 22.8283 | 112698 | 1173711 | NODE_62 | 1829 | 69.3267 | 303 | 1171225 |
| ag | NODE_14 | 114065 | 22.8283 | 112702 | 1173715 | NODE_62 | 1829 | 69.3267 | 307 | 1171229 |
| at | NODE_14 | 114065 | 22.8283 | 112704 | 1173717 | NODE_62 | 1829 | 69.3267 | 309 | 1171231 |
| ta | NODE_14 | 114065 | 22.8283 | 112705 | 1173718 | NODE_62 | 1829 | 69.3267 | 310 | 1171232 |
| gt | NODE_14 | 114065 | 22.8283 | 112707 | 1173720 | NODE_62 | 1829 | 69.3267 | 312 | 1171234 |
| ca | NODE_14 | 114065 | 22.8283 | 112708 | 1173721 | NODE_62 | 1829 | 69.3267 | 313 | 1171235 |
| ac | NODE_14 | 114065 | 22.8283 | 112709 | 1173722 | NODE_62 | 1829 | 69.3267 | 314 | 1171236 |
| ta | NODE_14 | 114065 | 22.8283 | 112711 | 1173724 | NODE_62 | 1829 | 69.3267 | 316 | 1171238 |
| ag | NODE_14 | 114065 | 22.8283 | 112717 | 1173730 | NODE_62 | 1829 | 69.3267 | 322 | 1171244 |
| tg | NODE_14 | 114065 | 22.8283 | 112718 | 1173731 | NODE_62 | 1829 | 69.3267 | 323 | 1171245 |
| ct | NODE_14 | 114065 | 22.8283 | 112721 | 1173734 | NODE_62 | 1829 | 69.3267 | 326 | 1171248 |
| tc | NODE_14 | 114065 | 22.8283 | 112723 | 1173736 | NODE_62 | 1829 | 69.3267 | 328 | 1171250 |
| gt | NODE_14 | 114065 | 22.8283 | 112724 | 1173737 | NODE_62 | 1829 | 69.3267 | 329 | 1171251 |
| ct | NODE_14 | 114065 | 22.8283 | 112729 | 1173742 | NODE_62 | 1829 | 69.3267 | 336 | 1171258 |
| gt | NODE_14 | 114065 | 22.8283 | 112731 | 1173744 | NODE_62 | 1829 | 69.3267 | 338 | 1171260 |
| ct | NODE_14 | 114065 | 22.8283 | 112732 | 1173745 | NODE_62 | 1829 | 69.3267 | 339 | 1171261 |
| ca | NODE_14 | 114065 | 22.8283 | 112733 | 1173746 | NODE_62 | 1829 | 69.3267 | 340 | 1171262 |
| ta | NODE_14 | 114065 | 22.8283 | 112734 | 1173747 | NODE_62 | 1829 | 69.3267 | 341 | 1171263 |
| ca | NODE_14 | 114065 | 22.8283 | 112739 | 1173752 | NODE_62 | 1829 | 69.3267 | 346 | 1171268 |
| at | NODE_14 | 114065 | 22.8283 | 112744 | 1173757 | NODE_62 | 1829 | 69.3267 | 350 | 1171272 |
| ga | NODE_14 | 114065 | 22.8283 | 112745 | 1173758 | NODE_62 | 1829 | 69.3267 | 351 | 1171273 |
| ct | NODE_14 | 114065 | 22.8283 | 112746 | 1173759 | NODE_62 | 1829 | 69.3267 | 352 | 1171274 |
| ac | NODE_14 | 114065 | 22.8283 | 112748 | 1173761 | NODE_62 | 1829 | 69.3267 | 354 | 1171276 |
| ag | NODE_14 | 114065 | 22.8283 | 112750 | 1173763 | NODE_62 | 1829 | 69.3267 | 356 | 1171278 |
| ag | NODE_14 | 114065 | 22.8283 | 112753 | 1173766 | NODE_62 | 1829 | 69.3267 | 359 | 1171281 |
| ac | NODE_14 | 114065 | 22.8283 | 112754 | 1173767 | NODE_62 | 1829 | 69.3267 | 360 | 1171282 |
| at | NODE_14 | 114065 | 22.8283 | 112755 | 1173768 | NODE_62 | 1829 | 69.3267 | 361 | 1171283 |
| ga | NODE_14 | 114065 | 22.8283 | 112758 | 1173771 | NODE_62 | 1829 | 69.3267 | 364 | 1171286 |
| ta | NODE_14 | 114065 | 22.8283 | 112759 | 1173772 | NODE_62 | 1829 | 69.3267 | 365 | 1171287 |
| gt | NODE_14 | 114065 | 22.8283 | 112760 | 1173773 | NODE_62 | 1829 | 69.3267 | 366 | 1171288 |
| at | NODE_14 | 114065 | 22.8283 | 112766 | 1173779 | NODE_62 | 1829 | 69.3267 | 377 | 1171299 |
| gt | NODE_14 | 114065 | 22.8283 | 112769 | 1173782 | NODE_62 | 1829 | 69.3267 | 380 | 1171302 |
| ct | NODE_14 | 114065 | 22.8283 | 112770 | 1173783 | NODE_62 | 1829 | 69.3267 | 381 | 1171303 |
| at | NODE_14 | 114065 | 22.8283 | 112772 | 1173785 | NODE_62 | 1829 | 69.3267 | 383 | 1171305 |
| cg | NODE_14 | 114065 | 22.8283 | 112773 | 1173786 | NODE_62 | 1829 | 69.3267 | 384 | 1171306 |
| ag | NODE_14 | 114065 | 22.8283 | 112774 | 1173787 | NODE_62 | 1829 | 69.3267 | 385 | 1171307 |
| at | NODE_14 | 114065 | 22.8283 | 112776 | 1173789 | NODE_62 | 1829 | 69.3267 | 387 | 1171309 |
| at | NODE_14 | 114065 | 22.8283 | 112780 | 1173793 | NODE_62 | 1829 | 69.3267 | 391 | 1171313 |
| gt | NODE_14 | 114065 | 22.8283 | 112782 | 1173795 | NODE_62 | 1829 | 69.3267 | 393 | 1171315 |
| gt | NODE_14 | 114065 | 22.8283 | 112787 | 1173800 | NODE_62 | 1829 | 69.3267 | 402 | 1171324 |
| tc | NODE_14 | 114065 | 22.8283 | 112788 | 1173801 | NODE_62 | 1829 | 69.3267 | 403 | 1171325 |
| tc | NODE_14 | 114065 | 22.8283 | 112789 | 1173802 | NODE_62 | 1829 | 69.3267 | 404 | 1171326 |
| ag | NODE_14 | 114065 | 22.8283 | 112790 | 1173803 | NODE_62 | 1829 | 69.3267 | 405 | 1171327 |
| ga | NODE_14 | 114065 | 22.8283 | 112793 | 1173806 | NODE_62 | 1829 | 69.3267 | 408 | 1171330 |
| tc | NODE_14 | 114065 | 22.8283 | 112795 | 1173808 | NODE_62 | 1829 | 69.3267 | 413 | 1171335 |
| ta | NODE_14 | 114065 | 22.8283 | 112798 | 1173811 | NODE_62 | 1829 | 69.3267 | 416 | 1171338 |
| ag | NODE_14 | 114065 | 22.8283 | 112800 | 1173813 | NODE_62 | 1829 | 69.3267 | 418 | 1171340 |
| ca | NODE_14 | 114065 | 22.8283 | 112801 | 1173814 | NODE_62 | 1829 | 69.3267 | 419 | 1171341 |
| ta | NODE_14 | 114065 | 22.8283 | 112802 | 1173815 | NODE_62 | 1829 | 69.3267 | 420 | 1171342 |
| ct | NODE_14 | 114065 | 22.8283 | 112804 | 1173817 | NODE_62 | 1829 | 69.3267 | 422 | 1171344 |
| ga | NODE_14 | 114065 | 22.8283 | 112805 | 1173818 | NODE_62 | 1829 | 69.3267 | 423 | 1171345 |
| tc | NODE_14 | 114065 | 22.8283 | 112807 | 1173820 | NODE_62 | 1829 | 69.3267 | 425 | 1171347 |
| tc | NODE_14 | 114065 | 22.8283 | 112810 | 1173823 | NODE_62 | 1829 | 69.3267 | 428 | 1171350 |
| ga | NODE_14 | 114065 | 22.8283 | 112811 | 1173824 | NODE_62 | 1829 | 69.3267 | 429 | 1171351 |
| ag | NODE_14 | 114065 | 22.8283 | 112812 | 1173825 | NODE_62 | 1829 | 69.3267 | 430 | 1171352 |
| gt | NODE_14 | 114065 | 22.8283 | 112818 | 1173831 | NODE_62 | 1829 | 69.3267 | 436 | 1171358 |
| ac | NODE_14 | 114065 | 22.8283 | 112821 | 1173834 | NODE_62 | 1829 | 69.3267 | 439 | 1171361 |
| ct | NODE_20 | 89542 | 23.3383 | 59629 | 1234707 | NODE_20 | 89653 | 72.4171 | 59740 | 1232491 |
| ga | NODE_20 | 89542 | 23.3383 | 59648 | 1234726 | NODE_20 | 89653 | 72.4171 | 59759 | 1232510 |
| tg | NODE_20 | 89542 | 23.3383 | 59719 | 1234797 | NODE_20 | 89653 | 72.4171 | 59830 | 1232581 |
| tc | NODE_20 | 89542 | 23.3383 | 59848 | 1234926 | NODE_20 | 89653 | 72.4171 | 59959 | 1232710 |
| tg | NODE_20 | 89542 | 23.3383 | 59849 | 1234927 | NODE_20 | 89653 | 72.4171 | 59960 | 1232711 |
| ta | NODE_20 | 89542 | 23.3383 | 59872 | 1234950 | NODE_20 | 89653 | 72.4171 | 59983 | 1232734 |
| ag | NODE_20 | 89542 | 23.3383 | 59912 | 1234990 | NODE_20 | 89653 | 72.4171 | 60023 | 1232774 |
| ag | NODE_20 | 89542 | 23.3383 | 59919 | 1234997 | NODE_20 | 89653 | 72.4171 | 60030 | 1232781 |
| ct | NODE_20 | 89542 | 23.3383 | 59928 | 1235006 | NODE_20 | 89653 | 72.4171 | 60039 | 1232790 |
| ga | NODE_20 | 89542 | 23.3383 | 59944 | 1235022 | NODE_20 | 89653 | 72.4171 | 60055 | 1232806 |
| ga | NODE_20 | 89542 | 23.3383 | 59950 | 1235028 | NODE_20 | 89653 | 72.4171 | 60061 | 1232812 |
| tg | NODE_20 | 89542 | 23.3383 | 59972 | 1235050 | NODE_20 | 89653 | 72.4171 | 60083 | 1232834 |
| at | NODE_20 | 89542 | 23.3383 | 60010 | 1235088 | NODE_20 | 89653 | 72.4171 | 60121 | 1232872 |
| ag | NODE_20 | 89542 | 23.3383 | 60446 | 1235524 | NODE_20 | 89653 | 72.4171 | 60557 | 1233308 |
| gt | NODE_15 | 112487 | 23.3897 | 620 | 3406523 | NODE_15 | 112486 | 81.5327 | 620 | 3404619 |
| tg | NODE_2 | 285698 | 23.2082 | 113054 | 4232928 | NODE_2 | 285898 | 80.8277 | 113254 | 4229015 |
| gc | NODE_55 | 3027 | 200.141 | 91 | 4562477 | NODE_55 | 3027 | 1014.71 | 91 | 4558564 |
| gc | NODE_55 | 3027 | 200.141 | 1277 | 4563663 | NODE_55 | 3027 | 1014.71 | 1277 | 4559750 |
| ga | NODE_55 | 3027 | 200.141 | 1278 | 4563664 | NODE_55 | 3027 | 1014.71 | 1278 | 4559751 |
| ct | NODE_55 | 3027 | 200.141 | 1285 | 4563671 | NODE_55 | 3027 | 1014.71 | 1285 | 4559758 |
| gt | NODE_57 | 2412 | 1.87527 | 31 | 4565444 | NODE_58 | 2109 | 3.57316 | 224 | 4561724 |
| at | NODE_57 | 2412 | 1.87527 | 90 | 4565503 | NODE_58 | 2109 | 3.57316 | 283 | 4561783 |
| at | NODE_57 | 2412 | 1.87527 | 1891 | 4567304 | NODE_58 | 2109 | 3.57316 | 2084 | 4563584 |
| tc | NODE_57 | 2412 | 1.87527 | 1893 | 4567306 | NODE_58 | 2109 | 3.57316 | 2086 | 4563586 |
| tc | NODE_57 | 2412 | 1.87527 | 1894 | 4567307 | NODE_58 | 2109 | 3.57316 | 2087 | 4563587 |
| ac | NODE_57 | 2412 | 1.87527 | 1898 | 4567311 | NODE_58 | 2109 | 3.57316 | 2091 | 4563591 |
| ta | NODE_57 | 2412 | 1.87527 | 1907 | 4567320 | NODE_58 | 2109 | 3.57316 | 2100 | 4563600 |
| tc | NODE_57 | 2412 | 1.87527 | 1909 | 4567322 | NODE_58 | 2109 | 3.57316 | 2102 | 4563602 |
| ta | NODE_57 | 2412 | 1.87527 | 1910 | 4567323 | NODE_58 | 2109 | 3.57316 | 2103 | 4563603 |
| ga | NODE_59 | 2073 | 48.1855 | 680 | 4568505 | NODE_59 | 2073 | 123.312 | 680 | 4564289 |
| tg | NODE_59 | 2073 | 48.1855 | 975 | 4568800 | NODE_59 | 2073 | 123.312 | 975 | 4564584 |
| tg | NODE_61 | 1345 | 59.8924 | 1 | 4569899 | NODE_60 | 1995 | 316.942 | 1 | 4565683 |
| cg | NODE_61 | 1345 | 59.8924 | 2 | 4569900 | NODE_60 | 1995 | 316.942 | 2 | 4565684 |
| ct | NODE_61 | 1345 | 59.8924 | 3 | 4569901 | NODE_60 | 1995 | 316.942 | 3 | 4565685 |
| ac | NODE_61 | 1345 | 59.8924 | 8 | 4569906 | NODE_60 | 1995 | 316.942 | 8 | 4565690 |
| gc | NODE_61 | 1345 | 59.8924 | 9 | 4569907 | NODE_60 | 1995 | 316.942 | 9 | 4565691 |
| cg | NODE_61 | 1345 | 59.8924 | 10 | 4569908 | NODE_60 | 1995 | 316.942 | 10 | 4565692 |
| ga | NODE_61 | 1345 | 59.8924 | 21 | 4569919 | NODE_60 | 1995 | 316.942 | 15 | 4565697 |
| ga | NODE_61 | 1345 | 59.8924 | 23 | 4569921 | NODE_60 | 1995 | 316.942 | 17 | 4565699 |
| ta | NODE_61 | 1345 | 59.8924 | 25 | 4569923 | NODE_60 | 1995 | 316.942 | 19 | 4565701 |
| tc | NODE_61 | 1345 | 59.8924 | 30 | 4569928 | NODE_60 | 1995 | 316.942 | 25 | 4565707 |
| ag | NODE_61 | 1345 | 59.8924 | 31 | 4569929 | NODE_60 | 1995 | 316.942 | 26 | 4565708 |
| tc | NODE_61 | 1345 | 59.8924 | 33 | 4569931 | NODE_60 | 1995 | 316.942 | 28 | 4565710 |
| cg | NODE_61 | 1345 | 59.8924 | 37 | 4569935 | NODE_60 | 1995 | 316.942 | 32 | 4565714 |
| cg | NODE_61 | 1345 | 59.8924 | 38 | 4569936 | NODE_60 | 1995 | 316.942 | 33 | 4565715 |
| tc | NODE_61 | 1345 | 59.8924 | 39 | 4569937 | NODE_60 | 1995 | 316.942 | 34 | 4565716 |
| tc | NODE_61 | 1345 | 59.8924 | 44 | 4569942 | NODE_60 | 1995 | 316.942 | 39 | 4565721 |
| cg | NODE_70 | 883 | 0.871693 | 319 | 4580401 | NODE_63 | 1732 | 1.22305 | 1375 | 4569052 |
| ca | NODE_70 | 883 | 0.871693 | 345 | 4580427 | NODE_63 | 1732 | 1.22305 | 1401 | 4569078 |

Supplementary Table 2. SNPS from table 1 above found to be in tRNA, rRNA or CDS regions from gbk files in the WT genome, all sequences hit (BLASTn) to *E.coli* sequences in Genbank with 100% id and query cover with no gaps (unless otherwise specified).

| Contig ID (WT) | SNP pos in contig | Annotated range in contig | Product | Blastp top hit and accession number |
| --- | --- | --- | --- | --- |
| NODE_14 | 1746 | 1330: 2118 | hypothetical protein | Phage Tail Collar Domain, ACX40129 |
| NODE_14 | 99500 | 98749: 100251 | Glucans biosynthesis protein G, mdoG | glucan biosynthesis protein G, mdoG,  EGX18964 |
| NODE_20 | 59629 | 59566:59673 | Small toxic polypeptide LdrD | Toxin Ldr, type I toxin-antitoxin system family protein, KQB23928 |
| NODE_15 | 620 | 333: 2447 | Elongation factor G | Elongation factor G EHO9441736 |
| NODE_2 | 113054 | 112915: 113217 | Inner membrane protein YjeO | YjeO family protein, WP_032360235 |
| NODE_59 | 680 | 673:1263 | Serine recombinase PinR | Recombinase family protein, MBC0929157 |
| NODE_70 | 345 | 329:670 | hypothetical protein | hypothetical protein, WP_050279308, Streptococcus pneumoniae, 93% identity. |

Supplementary Table 3. Protein products with SNPS from table 1 found to be in tRNA, rRNA or CDS regions from gbk files in the resistant (2mg/L) genome, all sequences hit (BLASTn) to *E.coli* sequences in Genbank with 100% id and query cover with no gaps.

| Contig ID | SNP pos in contig | Annotated range in contig | Product | Blastp top hit |
| --- | --- | --- | --- | --- |
| NODE_16 | 1746 | 1713:2186 | hypothetical protein | Phage portal protein [Escherichia coli] WP_001350502 (100%id and q cover) |
| NODE_16 | 99500 | 9689:10384 | Glucans biosynthesis protein G | Protein YmfE, phage or Prophage Related,  WP_010723094 |
| NODE_20 | 59629 | 60212:60319 | Small toxic polypeptide LdrD | Toxin Ldr, type I toxin-antitoxin system family protein,ERD00584 |
| NODE_15 | 620 | 333:2447 | Elongation factor G | Elongation factor G, HAI7289671 |
| NODE_2 | 113054 | 112915:113217 | Inner membrane protein YjeO | YjeO,WP_032360235 |
| NODE_59 | 680 | 673:1263 | Serine recombinase PinR | Recombinase family protein.HBH4704177 |
| NODE_63 | 345 | 306:839 | hypothetical protein | hypothetical protein, WP_235164902. |
